# A case report: Splenic abscess caused by *Burkholderia pseudomallei*

**DOI:** 10.1097/MD.0000000000011208

**Published:** 2018-06-29

**Authors:** Hai Chen, Zhi-qiang Hu, Yao Fang, Xiao-xue Lu, Li-da Li, Yuan-li Li, Xu-hu Mao, Qian Li

**Affiliations:** a Department of Clinical Laboratory, People's Hospital of Sanya, Sanya City, Hainan Province; b Department of Clinical Microbiology and Immunology, College of Medical Laboratory Science, Third Military Medical University (Army Medical University), Chongqing, China.

**Keywords:** *Burkholderia pseudomallei*, melioidosis, splenic abscess

## Abstract

**Rationale:**

Melioidosis is an emerging infectious disease caused by Burkholderia pseudomallei. To our knowledge, there have been very few cases of splenic abscesses due to melioidosis in Hainan, China.

**Patient concerns:**

The patient was a 55-year-old male farmer, who was admitted in our hospital with persistent left epigastric dull pain accompanied by chills and febrile. One month before, the patient presented with persistent abdominal pain. After received anti-infection therapy, the subjective symptoms eased slightly, but recently he suffered from intermittent abdominal pain again.

**Diagnoses:**

Bacteria isolated from splenic pus were identified as B. pseudomallei by the Phoenix-100 system and indirect immunofluorescence.

**Interventions:**

The patient was treated by surgical excision and anti-infection therapy.

**Outcomes:**

The patient was then treated with intravenous ceftazidime and oral trimethoprim-sulfamethoxazole for 2 weeks and his clinical symptoms improved.

**Lessons:**

In endemic areas, B. pseudomallei should be considered as a causative organism of splenic abscess in patients with established risk factors. The isolation of B. pseudomallei from abscess sites is crucial to improve clinical outcomes by appropriate antimicrobial therapy coupled with surgical drainage.

## 1 Introduction

Melioidosis is a severe systemic infectious disease caused by *Burkholderia pseudomallei*, a motile gram-negative bacillus with bipolar staining.^[1]^*B pseudomallei* is an environmental saprophyte that is commonly found in the soil, groundwater, rice paddies, and ponds throughout endemic regions.^[2]^ The disease is transmitted through contact with contaminated soil or water by percutaneous inoculation, aerosol inhalation, or the ingestion of contaminated water or food. The fatality rate of melioidosis ranges from 19% to 36% in endemic areas.^[3,4]^ Specific risk factors for the development of severe melioidosis are diabetes, thalassemia, renal disease (defined as renal calculi or renal failure), and occupational exposure to environmental contaminants.^[5,6]^ Clinical syndromes include subclinical infections, asymptomatic or minor localized abscesses, severe pneumonia, and fulminant sepsis.^[2]^ Splenic abscess is rare, even in endemic areas, with only a few reports in the literature. We present a case report of splenic abscess due to *B pseudomallei* infection.

## 2 Case report

A 55-year-old male farmer presented to the Outpatient Department with persistent left epigastric dull pain accompanied by chills and febrile (*T*_max_ 39 °C). He did not report a clear cause of the abdominal pain. An abdominal exam revealed no distended abdomen, normoactive bowel sounds, no rebound tenderness, no tenderness in the left upper quadrant, no palpable mass. He lost 10 kg within 6 months unintentionally. He visited a gastroenterologist at a health clinic and received anti-infection therapy, which relieved the abdominal pain. However, he recently suffered from intermittent abdominal pain. He was subsequently referred to our hospital for further evaluation and treatment.

**Figure 1:**
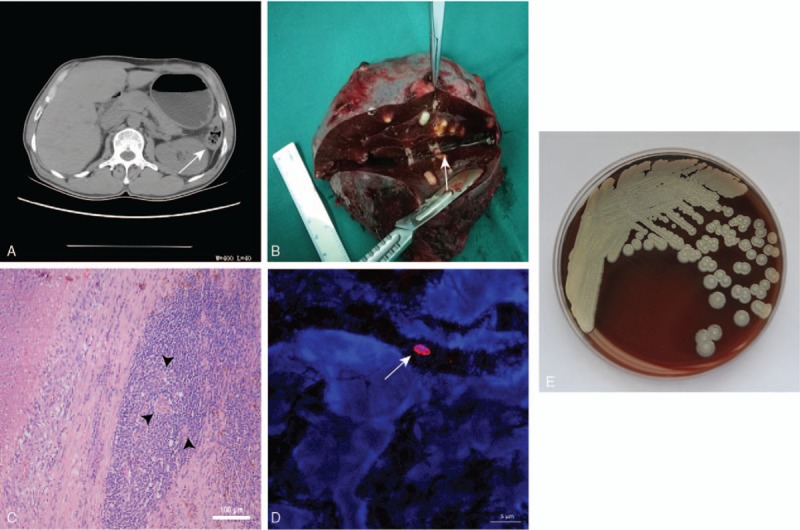
(A) Computed tomography images of the abdomen of the patient, splenic parenchyma, and subcapsular see multiple cystic low-density size (arrow), clear boundaries. (B) Cut section of the spleen showing caseous necrosis and many different sizes of pus cavities (arrow). (C) Granulomatous inflammation with a central area of necrosis surrounded by foamy histiocytes and epithelioid macrophages with scattered multinucleated giant cells (arrowhead). (D) Confocal microscopy image showing the morphology of the bacilli (arrow). (E) Bacterial colony morphology after 72 hours of pus culture in blood agar. Magnification: ×20 objective lens (C); ×63 objective lens (D).

The patient presented with fever and left upper abdominal pain. In a blood sample, the white blood cell count was 17.37 × 10^9^ cells/L (neutrophils = 93.9%, lymphocytes = 3.1%, monocytes = 3.0%, eosinophils = 0.00%, basophils = 0.00%) and the platelet count was 289 × 10^9^ cells/L when he first came to the outpatient department. The results of laboratory tests, including urine test, liver function tests, alpha-fetoprotein, carcinoembryonic antigen, and carbohydrate antigen 19–9, were all normal. Highly sensitive C-reactive protein was 56.6 mg/L (Table 1). He had a 1-year history of type 2 diabetes mellitus and took medicine irregularly. He had no hepatitis or tuberculosis, and a human immunodeficiency virus serological test was negative. Upper abdominal contrast-enhanced computed tomography (CT) showed diffuse hepatic disease, spleen abscess with inflammatory exudate, and spleen calcification (Fig. 1A). A chest radiograph and CT scan showed chronic inflammation of the inferior lobe of the left lung, a small amount of fluid in the left chest, and thickening of the wall of the gastric fundus.

**Table 1:**
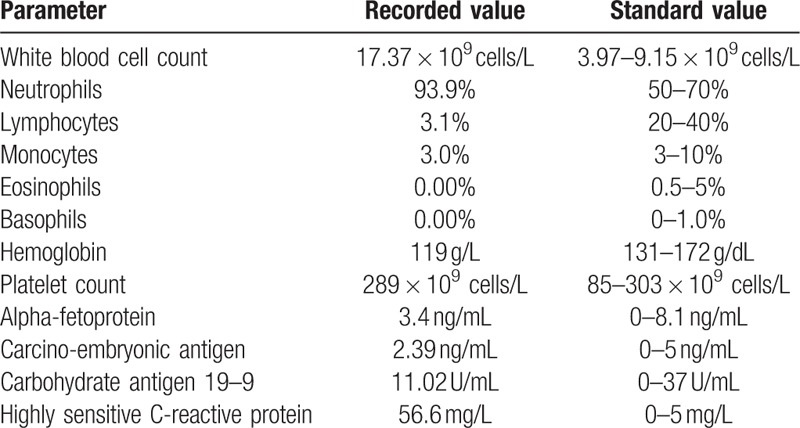
Laboratory data on admission.

The patient was subsequently subjected to laparoscopic exploration and splenectomy. A histological examination of biopsies demonstrated obvious spleen enlargement, fibrosis, and necrosis of spleen parenchyma, presenting as “frozen” lesions. Additionally, the tail of the pancreas and greater curvature of the stomach adhered to the spleen. A peritoneal cavity drainage tube was inserted in the splenic recess for the drainage of pus. A spleen specimen of 12 × 8 × 6 cm exhibited a number of divergent pus cavities, the largest of which had a diameter of 2 cm (Fig. 1B). Two days after surgery, the patient developed an effusion in the splenic fossa, which was successfully drained.

We suspected tuberculosis according to a histological analysis of HE-stained spleen sections. Typical granulomatous inflammation was observed in splenic pathological sections (Fig. 1C). However, the specific acid-fast staining test for tuberculosis was negative (data not shown). Immunohistochemical staining was performed using a *B pseudomallei* antibody preserved in our laboratory, and *B pseudomallei* in the splenic tissue sections were observed (Fig. 1D). Bacterial culture of the spleen pus for 72 hours revealed small, dry colonies with a typical wrinkled surface on blood agar (Fig. 1E). Bacteria isolated from the pus culture were identified as *B pseudomallei* using the commercial BD Phoenix-100 Automated Microbiology System (BD Biosciences, Franklin Lakes, NJ). Based on analyses of the minimum inhibitory concentration (MIC), the isolated strain was susceptible to ceftazidime (MIC, 4 μg/mL), meropenem (MIC, 2 μg/mL), and trimethoprim-sulfamethoxazole (TMP-SMX) (MIC, 0.5 μg/mL).

The patient was treated with antibiotics intravenously for 15 days. On the day of admission, he initially received empirical anti-infective intravenous cefmenoxime and piperacillin-tazobactam. Owing to the lack of a clinical response, the treatment was changed to intravenous ceftazidime for 2 days, followed by oral TMP-SMX according to the results of a drug sensitivity test, and the patient exhibited a gradual improvement in clinical condition. Upper abdominal CT showed minor pneumoperitoneum and a reduction in pleural fluid in the left lung lobe. The pigtail was removed after no further abscess drainage was necessary. The patient was asymptomatic for the next 5 days, and the wound was clean and granulating. Hence, he was discharged with oral TMP-SMX for 3 months for eradication therapy.

## 3 Discussion

Melioidosis is an emerging infectious disease caused by the non-spore-forming, gram-negative soil saprophytic bacillus *B pseudomallei*. Although melioidosis mainly occurs in Southeast Asia and northern Australia, it has been increasingly reported outside of the Asia-Pacific region, for example, in China,^[7,8]^ India,^[9]^ and the Americas.^[10]^ The clinical manifestations of melioidosis can include localized soft tissue lesions, visceral abscesses (in the spleen, lung, and liver), and fulminant septicemia. However, the most frequent presentation is septicemia and community-acquired pneumonia.^[11]^ Affected individuals usually have well-known predisposing factors, such as diabetes mellitus, chronic lung, and renal disease, or are immunocompromised.^[11]^ The disease is acquired from contact with contaminated soil or water through abrasions and minor cuts, as well as by the inhalation of aerosols and ingestion.

Splenic abscesses are not commonly associated with melioidosis. To our knowledge, there have been very few cases of splenic abscesses due to melioidosis in Hainan.^[7]^ In Singapore, the most common etiological agent was *B pseudomallei* according to a recent study.^[12]^ In this case study, the patient had no history of traveling abroad and no history of tuberculosis or hepatitis. However, he had a history of uncontrolled diabetes for 1 year and had a knife wound on the right back 2 years ago. We were unable to establish a connection between the splenic abscess and the knife wound, so the transmission route is unknown. Pre-existing diabetes mellitus and wound inoculation could hypothetically contribute to an increased susceptibility to melioidosis. A delay in clinical diagnosis could be lethal because *B pseudomallei* harbor the *amrAB-oprA* efflux pump. It is naturally resistant to aminoglycosides and zeocin. Antimicrobial therapy with susceptible agents (ceftazidime and TMP-SMX) is usually effective. In this case, the patient was treated by splenectomy for pyogenic splenic abscess, since for visceral abscesses caused by melioidosis, surgical intervention and percutaneous drainage with recommended antibiotics have both shown good outcomes and a low rate of relapse.

Hainan is a major area of melioidosis in China. As a result of increased international travel, the disease incidence has increased year-by-year, according to our retrospective study of melioidosis cases over a recent 11-year period (2002–2013) in Hainan.^[7]^ The prompt diagnosis of infection and adequate antibiotic treatment are crucial determinants of disease outcomes. The isolation of *B pseudomallei* from clinical specimens is considered the “gold standard” for melioidosis diagnosis. In this case, a high index of clinical suspicion combined with indirect immunoinfluscent assay and bacterial culture contributed to the diagnosis. The blood culture was initially negative but subsequent splenic pus was confirmed to be positive for *B pseudomallei* by culture and the Automatic Bacteria Identification System. These results suggested that *B pseudomallei* should be considered a causative agents of organ abscess in any patient with risk factors in endemic areas after other illnesses are excluded. Improving awareness of *B pseudomallei* may reduce mortality and morbidity among the high-risk group with a history of diabetes and outdoor labor.

## 4 Conclusions

*B pseudomallei* is a potential causative organism of splenic abscess in patients with risk factors who reside in endemic areas and do not respond to standard antibiotics. A pus culture from the infection site is the only diagnostic method for visceral abscess melioidosis. Appropriate antibiotics and adequate surgical drainage can contribute to a successful outcome.

## Author contributions

**Conceptualization:** Qian Li.

**Data curation:** Hai Chen, Li-da Li, Yuan-li Li, Xu-hu Mao.

**Formal analysis:** Yao Fang.

**Project administration:** Xiao-xue Lu.

**Writing – original draft:** Zhi-qiang Hu.
